# Nanobiosensors for Single-Molecule Diagnostics: Toward Integration with Super-Resolution Imaging

**DOI:** 10.3390/bios15100705

**Published:** 2025-10-21

**Authors:** Seungah Lee, Sobia Rafiq, Seong Ho Kang

**Affiliations:** 1 Department of Applied Chemistry and Institute of Natural Sciences, Kyung Hee University, Yongin-si 17104, Gyeonggi-do, Republic of Korea; salee@khu.ac.kr; 2 Department of Chemistry, Graduate School, Kyung Hee University, Yongin-si 17104, Gyeonggi-do, Republic of Korea; sobiarafiq@khu.ac.kr

**Keywords:** nanobiosensor, single-molecule detection, super-resolution microscopy, diagnostics

## Abstract

Recent advances in nanotechnology and optical imaging have transformed molecular diagnostics, enabling the detection and analysis of individual biomolecules with unprecedented precision. Nanobiosensors provide ultrasensitive molecular detection, and super-resolution microscopy (SRM) exceeds the diffraction limit of conventional optics to achieve nanometer-scale resolution. Although their integration remains in its infancy, with only a handful of proof-of-concept studies reported, the convergence of nanobiosensors and SRM holds significant promise for next-generation diagnostics. In this review, we first outline nanobiosensor-based single-molecule detection strategies and highlight representative implementations. These include plasmonic–SRM hybrids, electrochemical–optical correlatives, and SRM-enabled immunoassays, with a focus on their applications in oncology, infectious diseases, and neurodegenerative disorders. Then, we discuss emerging studies at the interface of nanobiosensors and SRM, including nanostructure-assisted SRM. Despite not being true biosensing approaches, these studies provide valuable insights into how engineered nanomaterials can improve imaging performance. Finally, we evaluate current challenges, including reproducibility, multiplexing, and clinical translation, and outline future opportunities, such as the development of photostable probes, artificial intelligence-assisted image reconstruction, microfluidic integration, and regulatory strategies. This review highlights the synergistic potential of nanobiosensors and SRM, outlining a roadmap toward clinically translatable next-generation single-molecule diagnostic platforms.

## 1. Introduction

Accurate and ultrasensitive detection of disease-related biomolecules such as proteins, nucleic acids, and metabolites has become increasingly crucial for personalized medicine and precision diagnostics. Single-molecule detection (SMD) technologies are central to achieving this objective [1]. Conventional assays, including enzyme-linked immunosorbent assays (ELISA), polymerase chain reaction (PCR), mass spectrometry, chromatography, and electrochemical methods, are extensively used to quantify biomarker levels in clinical and laboratory settings. However, despite being effective, these techniques typically fall short in detecting low-abundance biomarkers, particularly in complex biological fluids, such as peripheral blood [2,3]. Such limitations have spurred the development of advanced imaging approaches, particularly single-molecule and super-resolution microscopy (SRM) techniques, enabling the visualization and quantification of individual molecules with nanometer precision [4].

Various SMD assays, both labeled and label-free, have been developed to achieve nanometer-scale resolution of individual molecules. Labeled approaches include single-molecule localization microscopy, single-particle tracking, single-molecule Forster resonance energy transfer (smFRET), and single-molecule polarization imaging. These techniques reveal structural and kinetic heterogeneity typically obscured by ensemble-based methods, providing transformative insights into molecular biology, electrochemistry, materials science, and pharmaceutical research [4,5,6]. Alongside these fluorescence-based assays, label-free plasmonic methods such as surface plasmon resonance (SPR), localized surface plasmon resonance (LSPR), plasmonic scattering microscopy, and surface-enhanced Raman spectroscopy (SERS) enable real-time SMD and kinetic analysis at metal interfaces. These complement fluorescence by allowing mass sensing (LSPR/SPR) and molecular fingerprinting (SERS) [7,8,9].

In parallel, nanobiosensors, which are analytical devices integrating nanotechnology with biological recognition, have demonstrated remarkable advantages over conventional sensing methods for detecting diverse biological and chemical entities [10,11,12]. By leveraging the high surface-to-volume ratio, quantum confinement effects, and enhanced reactivity of nanomaterials such as nanoparticles (NPs), nanotubes, nanowires, and nanopores, these sensors achieve outstanding sensitivity and specificity. Their applications span from environmental monitoring to clinical diagnostics, with particular importance for the early detection of cancer, infectious diseases, and neurodegenerative disorders [13,14,15]. The integration of nanobiosensors with SRM platforms enables spatially resolved interrogation of biomolecular interactions beyond the diffraction limit of conventional optics.

In recent years, numerous reviews have addressed nanobiosensors or SRM techniques individually. However, there remains a lack of comprehensive summaries focusing on their integration. In this review, we first provide an overview of nanobiosensors, including their design principles, components, and detection strategies. Then, we examine SMD approaches and highlight how their convergence with SRM techniques, including stochastic optical reconstruction microscopy (STORM), photoactivated localization microscopy (PALM), stimulated emission depletion (STED), minimal photon fluxes (MINFLUX) nanoscopy, and DNA points accumulation for imaging in nanoscale topography (DNA-PAINT), has advanced biomolecular diagnostics. Next, we discuss representative applications, such as plasmonic–SRM hybrids, electrochemical–optical correlatives, and SRM-enabled immunoassays, focusing on their implications for early disease detection and precision medicine. Finally, we evaluate current challenges, including reproducibility, multiplexing, and clinical translation, and outline emerging directions, such as scalable fabrication, photostable probes, artificial intelligence (AI)-assisted image reconstruction, microfluidic integration, and regulatory considerations. Unlike previous reviews that have primarily focused on nanobiosensors or SRM individually, this study provides an integrated perspective, highlighting the transformative potential of nanobiosensor–SRM synergy for next-generation molecular diagnostics. Foundational studies on plasmonic biosensing [16,17,18] and pioneering SRM breakthroughs [19,20,21] have established the conceptual basis for the subsequent integration of nanobiosensors and SRM techniques. These studies have laid the groundwork for the rapid progress observed in recent years.

## 2. Nanobiosensors: Overview

### 2.1. Biosensor

Biosensors are analytical devices that detect and quantify specific biological and molecular compounds from biological samples. Their core function is to convert molecular recognition or binding events into measurable physical signals. Biosensors provide rapid, accurate, real-time, and reliable information about the analyte of interest. The first biosensor was introduced by Leland C. Clark, Jr. and Champ Lyons in 1962 [22,23]. Since then, the field has witnessed remarkable progress, with the development of innovative biosensor designs and continual improvements in sensitivity, selectivity, and portability.

### 2.2. Nanobiosensors

Nanobiosensors, which are developed using advanced nanotechnology techniques, are a class of sensors for observing, measuring, and analyzing biological events at the molecular level. These sensors incorporate various engineered nanomaterials, such as quantum dots (QDs), metallic and oxide nanoparticles (NPs), carbon-based nanowires (CNWs), and ultra-thin nanofilms, as functional components to improve signal generation and amplification. Nanoscale engineering of biosensors leverages the unique physicochemical properties of materials, including enhanced electron transport, plasmonic resonance, fluorescence yield, and surface interaction dynamics, to considerably improve performance. These enhancements are pivotal for detecting rare or low-abundance biomolecular targets with high sensitivity [10,24,25]. In addition to material design, the performance of nanobiosensors is typically evaluated using key analytical metrics, including limit of detection (LOD), sensitivity, selectivity, dynamic range, response time, and reproducibility. These parameters are strongly influenced by the choice of nanomaterial, surface functionalization strategy, and the transduction method employed, thereby determining the clinical and point-of-care (POC) applicability of the sensor.

#### 2.2.1. Working Principle

Nanobiosensors operate via specific interactions between the target analytes and bioreceptors, which induce detectable changes in the physicochemical, electrical, optical, thermal, or mechanical properties of the sensor. Nanomaterials serve as high-performance interfaces between the recognition elements and the transducer, improving sensitivity through their large surface-to-volume ratio, tunable optical/electronic characteristics, and quantum confinement effects. The transducer then converts these interactions into measurable signals such as electrical fluctuations, optical shifts, or resonance frequency changes, which are subsequently amplified, processed, and displayed by the readout system [26,27,28].

#### 2.2.2. Nanomaterials

Nanostructures, including nanoclusters, nanorods, nanotubes, and nanowires, typically range from 1 to 100 nm. Their synthesis depends on parameters such as the precursor concentration, temperature, and processing time. The synthesis can follow either a top-down approach (e.g., laser ablation, arc discharge, physical vapor deposition, and ball milling) or a bottom-up approach (e.g., hydrothermal processing, chemical vapor deposition, sol–gel, and co-precipitation) [29,30]. They can also be fabricated biologically using eco-friendly agents such as plants, bacteria, fungi, algae, and biomimetic materials to yield biocompatible and low-toxicity nanostructures [31,32].

Common nanomaterials used in biosensors include gold NPs (AuNPs), graphene, and metal oxides, owing to their excellent conductivity, optical tunability, and catalytic properties. AuNPs are valued for their biocompatibility, and carbon nanotubes (CNTs) exhibit superior electrical conductivity [33,34,35,36]. Advances in materials science continue to drive progress in nanobiosensor technology. For enhanced specificity, NPs are typically functionalized with enzymes, antibodies, or nucleic acids to enhance molecular recognition.

In addition to these well-established materials, emerging two-dimensional (2D) nanomaterials, such as molybdenum disulfide and transition-metal carbides/nitrides (MXenes), and DNA origami-based nanostructures have expanded the toolbox of nanobiosensor platforms. These next-generation materials provide tunable surface chemistry, strong plasmonic or catalytic activity, and excellent biocompatibility, enabling ultrasensitive and selective biosensing applications. Nanomaterials can also be categorized by dimensionality: zero-dimensional (0D) structures such as fullerenes, one-dimensional structures such as CNTs, 2D materials such as graphene, and three-dimensional (3D) structures such as graphite [31,32]. Nanobiosensors provide a versatile platform that bridges nanotechnology and molecular biology, providing unprecedented opportunities for early disease detection, environmental monitoring, and precision diagnostics.

### 2.3. Microfluidics and Nanobiosensors

Recent advances have successfully integrated nanobiosensors with portable analytical platforms, particularly microfluidic devices, to enhance on-site detection capabilities. Microfluidic systems offer several advantages, including simultaneous processing of multiple samples, high throughput, short analysis times, and minimal reagent and specimen consumption [9].

Microfluidic platforms allow highly controlled sensing processes in lab-on-a-chip (LOC) configurations by enabling precise manipulation of minute fluid volumes within lithographically fabricated microchannels. These systems can also replicate cell culture microenvironments and facilitate the isolation of microparticles such as extracellular vesicles (EVs). For example, a microfluidic-integrated biosensor was developed for breast cancer diagnostics by targeting EV-associated microRNA biomarkers. This LOC device exhibited an impressive LOD of 84 aM, with a dynamic range of 1 fM–1 nM [37]. Similar approaches have been extended to infectious disease diagnostics, such as SARS-CoV-2 (severe acute respiratory syndrome coronavirus 2) antigen and nucleic acid detection, and to single-cell analysis platforms in which microfluidics provides rapid cell sorting and high-throughput molecular profiling [38,39]. Therefore, the integration of nanobiosensors with microfluidic technologies not only enhances analytical sensitivity and throughput but also enables sample preprocessing, multiplexing, and automation within a single device. Such platforms are emerging as promising candidates for POC testing. They can be further advanced by coupling with smartphone-based readout systems and wearable LOC devices, highlighting their transformative potential for clinical translation and personalized diagnostics.

### 2.4. Surface Functionalization

Surface functionalization considerably influences biosensor performance by enabling the selective and stable immobilization of bioreceptors onto nanomaterials. Functional groups such as –COOH, –NH_2_, and –SH, along with polymer coatings such as polyethylene glycol (PEG) and polydopamine, are extensively used to improve biocompatibility, minimize nonspecific binding, and support ultrasensitive detection. Molecularly imprinted polymers (MIPs) have also been used to provide synthetic recognition sites with high stability and low production costs [40,41].

A critical challenge in nanomaterial-based biosensors is the tendency of NPs to aggregate or lose dispersibility under physiological or environmental stress. Recent studies have demonstrated effective strategies to overcome this limitation. For example, carboxylate-terminated ligands have been used to stabilize AuNPs under diverse pH conditions and high ionic strength while enabling facile conjugation with antibodies for pathogen detection [42]. Similarly, dual functionalization approaches, such as combining aptamers with PEG layers on graphene field-effect transistors (GFETs), have achieved both high specificity and reduced nonspecific adsorption, enabling picomolar-level detection of cytokines in complex physiological media [43].

In addition to these conventional approaches, emerging functionalization strategies have been used to exploit advanced nanostructures and bio interfaces. Zwitterionic coatings and click-chemistry-based linkers provide enhanced antifouling properties, improving sensor performance in whole-blood or serum samples [44]. DNA origami nanostructures and peptide-based linkers provide programmable biomolecule spacing and orientation, optimizing recognition efficiency and signal reproducibility [45]. In addition, hybrid interfaces that integrate inorganic nanomaterials with biomimetic membranes are gaining traction for applications requiring long-term stability and real-time monitoring in physiological environments [46].

Surface functionalization is a cornerstone in nanobiosensor design. The continuous development of robust, antifouling, and multifunctional coatings is expected to accelerate the clinical translation of these devices, ensuring reproducibility and reliability across diverse biomedical and environmental applications.

### 2.5. Nanobiosensor Components

Nanobiosensors typically comprise four major components: (i) bioreceptors, (ii) transducers, (iii) electronic systems, and (iv) display/readout modules. Each component plays a critical role in determining the sensitivity, selectivity, and overall performance of the biosensor.

Bioreceptors provide the molecular recognition element that imparts specificity toward the target analyte. Conventional recognition elements include antibodies, enzymes, nucleic acids, and aptamers [47]. More recently, synthetic recognition platforms such as MIPs, nanobodies, and whole-cell systems have emerged as promising alternatives because of their enhanced stability, reproducibility, and cost-effectiveness [10,48]. Transducers convert the recognition event into a measurable physical signal. Depending on the modality, transducers may operate via optical mechanisms (e.g., fluorescence and plasmonic resonance), electrical or electrochemical mechanisms [e.g., impedimetry/electrochemical impedance spectroscopy and field-effect transistor (FET) sensing], or thermal, acoustic, and magnetic mechanisms [49,50,51]. Optical transducers are favored for label-free, real-time monitoring; electrochemical systems enable miniaturization and low-cost POC platforms; thermal and acoustic designs suit enzyme activity or mass-based sensing; magnetic approaches provide excellent performance in complex matrices with reduced background noise. Electronic circuits amplify, process, and filter the raw signals generated by transducers to ensure accurate and high-precision readouts [52]. The miniaturization of electronic systems has allowed nanobiosensors to be integrated into portable and wearable devices. Recent progress in wireless electronics and smartphone-based interfaces has further enabled real-time monitoring and remote healthcare applications. Display and readout systems translate processed signals into user-friendly formats, enabling clinicians and end-users to rapidly interpret results. Conventional formats include optical density readings and electrical output values. However, modern platforms increasingly employ digital interfaces, cloud-based storage, and AI-driven data analysis pipelines to improve decision making and clinical utility [53].

Beyond their structural components, nanobiosensors can be classified according to their transduction mechanisms, which convert biochemical recognition events into measurable physical signals. The most commonly employed approaches include optical, electrochemical, thermal, mechanical, and magnetic modalities, each providing distinct advantages and limitations [54,55]. Optical transduction, encompassing fluorescence, LSPR, SERS, and SPR, provides high sensitivity, label-free detection, and strong multiplexing ability. However, this approach can be hindered by photobleaching and background interference. Electrochemical platforms, including amperometric, impedimetric, and FET-based sensors, are attractive for their miniaturization, portability, and low cost. However, they are susceptible to fouling in complex biological fluids [50]. Thermal and mechanical systems, such as calorimetric sensors, microcantilevers, and quartz crystal microbalance (QCM), enable direct mass or energy detection; however, they are vulnerable to environmental noise and require careful calibration [56]. Magnetic transduction, typically employing magnetic NPs or giant magnetoresistance devices (GMR), is effective for turbid samples with high signal-to-noise ratios; however, its performance depends on external magnetic fields and limited probe diversity [57].

## 3. SMD Biosensors

In response to the demand for highly sensitive biomedical diagnostics, the latest generation of nanobiosensors has advanced to achieve biomarker SMD. These platforms leverage nanoscale materials (e.g., NPs and 2D materials) and confined reaction volumes to convert individual binding events into detectable signals. Furthermore, seminal studies on LSPR/SPR biosensors [16,18] and early demonstrations of single-molecule SERS [58,59] have established the foundation for current nanoscale sensing strategies. The following sections summarize recent experimental progress across various nanobiosensor classes, including electrochemical, optical, plasmonic, SERS-based, hybrid, and nanoimmunosensors, highlighting their detection mechanisms, material platforms, and analytical performance in terms of sensitivity and LOD in biomedical applications [60].

**Figure 1:**
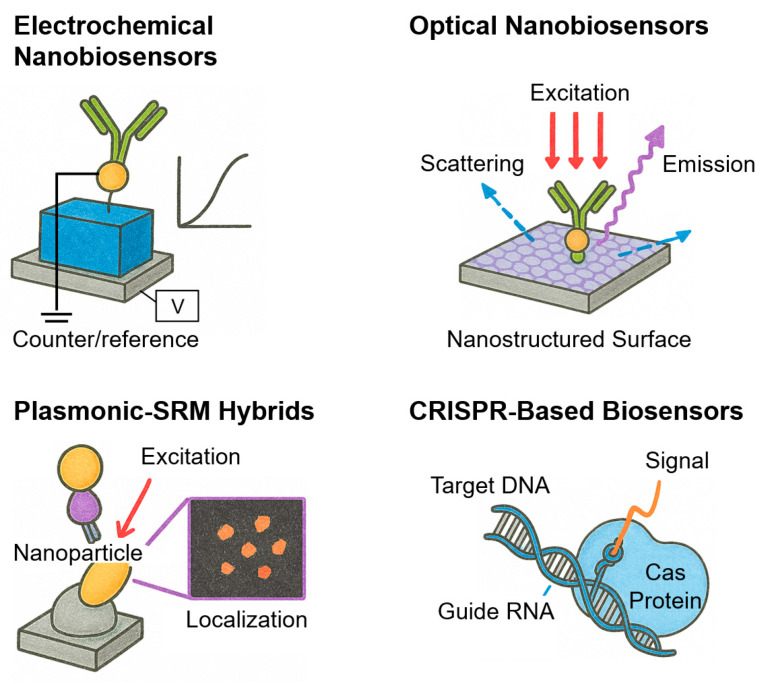
Schematic of four representative nanobiosensor modalities for single-molecule diagnostics. Electrochemical nanobiosensors detect single binding events via current or voltage shifts at nanostructured electrodes, enabling label-free and miniaturized sensing. Optical nanobiosensors monitor fluorescence, scattering, or refractive index changes amplified by nanostructured or photonic surfaces for ultrasensitive molecular recognition. Plasmonic–SRM hybrids integrate metallic nanostructures and SRM, combining plasmonic near-field enhancement and nanoscale localization precision. CRISPR-based biosensors exploit CRISPR-associated (Cas) proteins and guide RNAs to achieve highly specific nucleic acid detection via collateral cleavage of reporter probes.

Figure 1 shows a comparative schematic overview of the four representative classes of nanobiosensors discussed in this section. Electrochemical nanobiosensors transduce biomolecular binding into electrical signals via FETs or organic electrochemical transistors (OECTs). Optical nanobiosensors exploit photonic structures such as photonic crystals (PCs) and whispering-gallery mode (WGM) resonators to amplify fluorescence for single-molecule readout. Plasmonic–SRM hybrids integrate noble metal nanostructures and super-resolution imaging to achieve nanoscale mapping and enhanced localization precision. CRISPR-based biosensors use Cas12a-mediated trans-cleavage coupled with nanomaterial-assisted FRET for programmable nucleic acid detection. These platforms exemplify the diverse strategies by which nanobiosensors achieve single-molecule diagnostics. Although only four representative nanobiosensor classes (electrochemical, optical, plasmonic–SRM, and CRISPR-based) are shown in Figure 1, additional emerging modalities such as fluorescent nanoprobe–SRM platforms (Section 3.5) and hybrid/multimodal devices (Section 3.6) further expand the landscape of single-molecule diagnostics.

### 3.1. Electrochemical Nanobiosensors

Electrochemical nanobiosensors operate by monitoring changes in electrical properties, such as current, voltage, and impedance, that are triggered by biomolecular recognition events on electrode surfaces. This strategy offers several advantages: label-free detection, straightforward instrumentation, high sensitivity, miniaturization potential, and seamless integration with portable devices [61,62]. The incorporation of nanostructured electrodes (e.g., CNTs, graphene, and metal NPs) enhances electron transfer kinetics and the signal-to-noise ratio, thereby enabling detection down to the level of single binding events [63,64]. Notable examples include single-molecule electrochemical assays and FET-based biosensors for DNA and protein detection [65,66].

**Figure 2:**
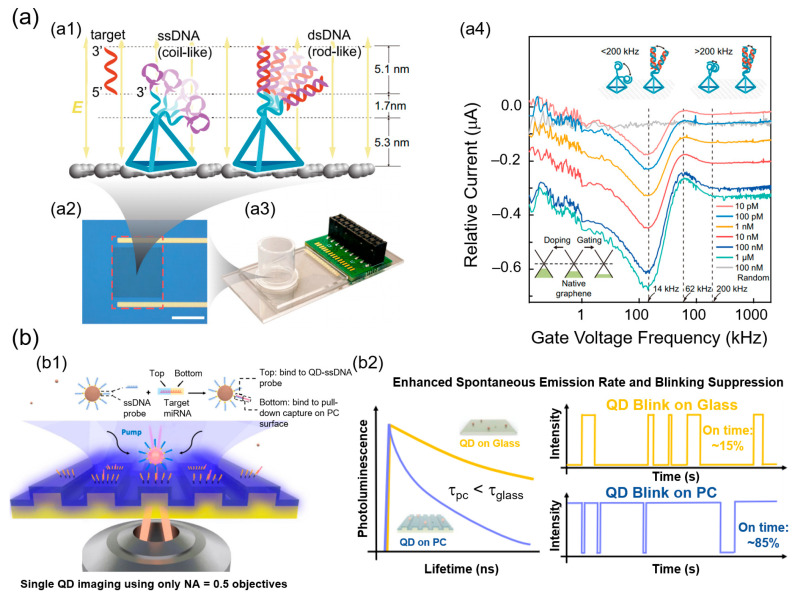
Electrochemical and optical nanobiosensor platforms for single-molecule diagnostics. (**a**) GFET-based electrochemical nanobiosensor. (**a1**) Tetrahedral DNA nanostructure anchors ssDNA probe (coil-like, purple) on the graphene surface. Upon hybridization with the complementary target strand (red), the probe forms rigid dsDNA (rod-like), which oscillates differently under the applied alternating electric field. (**a2**) The insets show the graphene sensing region (red box), and (**a3**) shows the integrated measurement system. (**a4**) Relative current spectra of GFET exhibiting frequency shifts with increasing target DNA concentration. The insets illustrate ssDNA (<200 kHz) vs. dsDNA (>200 kHz) oscillations and the graphene band diagram with Fermi level shifts. Reprinted with permission from [68]. Copyright (2023) National Academy of Sciences. (**b**) Optical nanobiosensor using PC substrate for single-molecule QD detection. (**b1**) Target miRNA binds QD–ssDNA probe (top) to capture probe on PC surface (bottom). (**b2**) The PC enhances spontaneous emissions and suppresses blinking, resulting in shorter lifetimes (τ_PC_ < τ_glass_) and higher on-time (~85%) than those obtained by QDs on glass (~15%). Reprinted with permission from [75], Copyright (2022) Nature Portfolio.

Recent advances have considerably expanded the capabilities of electrochemical nanobiosensors. For example, OECTs functionalized with nanobody probes enabled the direct detection of viral antigens for COVID-19 and MERS in unprocessed saliva and serum samples. This label-free OECT immunosensor achieved attomolar LODs, with a dynamic range spanning 8–10 orders of magnitude, and exhibited clinical performance comparable to RT-PCR (reverse transcription polymerase chain reaction) [67]. Similarly, GFETs have driven DNA analysis into the single-molecule range. In a previous study, DNA probes were immobilized onto a GFET and subjected to an alternating electric field, thereby inducing oscillatory strand motion. The distinct oscillation spectra allowed the discrimination of the single hybridization events, thereby improving sensitivity by two orders of magnitude compared to conventional FETs. As shown in Figure 2a, the tetrahedral DNA nanostructure anchored ssDNA probes on the graphene surface, and upon hybridization with complementary strands, these probes formed rigid dsDNA that oscillated differently under the applied field. This produced obvious frequency shifts in the current spectra, enabling femtomolar to sub-femtomolar quantification via direct electronic readout [68].

Electrochemical nanobiosensors provide a versatile and scalable platform for single-molecule diagnostics. Their combination of ultra-high sensitivity with miniaturized and low-cost architectures makes them particularly well suited for POC applications, viral diagnostics, and genetic screening. In addition, integrating electrochemical nanobiosensors with SRM techniques enables correlated analysis by linking electrical readouts with nanoscale spatial localization of molecular events, thereby enhancing mechanistic understanding and assay reproducibility. Despite their high sensitivity, electrochemical nanobiosensors still face challenges, including batch-to-batch variability in electrode nanostructures, surface fouling in complex biological samples, and poor long-term reproducibility. These limitations significantly hinder clinical translation, as standardization across laboratories remains difficult. Without scalable and reproducible nanofabrication methods, their promising potential for POC diagnostics may remain limited to proof-of-concept demonstrations.

### 3.2. Optical Nanobiosensors

Optical nanobiosensors detect molecular interactions by monitoring changes in the refractive index, fluorescence emissions, or light scattering that are triggered when biomolecules interact with a nanostructured surface or photonic device. These systems are highly attractive because they provide exceptional sensitivity, real-time monitoring, and the possibility of label-free operation [69,70]. Advanced optical strategies, including WGM resonators [71], PC sensors [72], and interferometric scattering microscopy (iSCAT) [73], have enabled the detection of single biomolecular binding events by capturing resonance shifts or discrete fluorescence bursts.

A notable example is the study of Yu et al. (2016), who developed a label-free optical nano-sensing strategy that exploited the optical spring effect in a high-Q coherent optomechanical oscillator. In this system, light circulates within a micro/nano-photonic cavity coupled to mechanical vibrations; molecular binding induces measurable resonance shifts amplified by changes in optomechanical stiffness. This approach substantially enhanced sensitivity compared with conventional cavity resonance sensing and enabled the SMD of bovine serum albumin (BSA, 66 kDa) with a signal-to-noise ratio of 16.8, without the need for fluorescent labeling [74].

PC platforms provide another powerful approach for ultrasensitive single-molecule analysis. By amplifying fluorescence emissions within confined optical modes, PC devices enable digital counting of single molecules. For example, a PC chip reported in 2022 improved the QD emission by approximately 3000-fold, thereby enabling high-fidelity single-molecule imaging. The chip also achieved a LOD of 10 aM for cancer-associated microRNA (miRNA) biomarkers, along with the observation of altered QD surface motion trajectories upon sequence variation. As shown in Figure 2b, the target miRNA hybridized a QD–ssDNA probe to a capture probe on a PC surface. The PC substrate enhanced spontaneous emissions and suppressed blinking. This resulted in shorter fluorescence lifetimes and markedly higher on-time ratios (~85%) compared with QDs on glass (~15%), providing reliable single-molecule resolution for ultra-low-concentration miRNA detection [75].

Optical nanobiosensors, including WGM resonators, PC devices, and iSCAT microscopy, offer powerful label-free or fluorescence-enhanced approaches for SMD. Their integration with SRM combines ultrasensitive optical sensing with nanoscale spatial resolution, enabling quantitative and spatially resolved single-molecule diagnostics. Despite these advantages, optical nanobiosensors remain limited by several intrinsic constraints. Photobleaching, labeling requirements, and inconsistent fluorescence stability across different substrates continue to impede reproducibility, particularly in live-cell and in vivo environments. Furthermore, the complexity of optical alignment and reliance on expensive photonic chips restrict their scalability and integration into portable or POC systems. Consequently, their use remains largely confined to specialized laboratories rather than routine clinical diagnostics.

For example, STORM and PALM combined with nanostructured sensing substrates have enabled nanoscale mapping of protein–DNA interactions, revealing spatial heterogeneity at the single-molecule level [76]. Similarly, DNA-PAINT coupled with QD-based probes has been used to achieve multiplexed detection of nucleic acids with nanometer precision [77]. Thus, these SRM-enabled optical nanobiosensors provide both quantitative and spatially resolved insights, overcoming the limitations of ensemble fluorescence assays.

### 3.3. Plasmonic–SRM Hybrids

Beyond nucleic acids and proteins, plasmonic nanostructures have also been employed for exosome biosensing, enabling the highly sensitive detection of EVs with direct relevance to cancer diagnostics [78]. Plasmonic nanobiosensors exploit the interactions between light and metallic nanostructures to generate collective electron oscillations known as surface plasmons, which are highly sensitive to changes in the local dielectric environment [79]. Depending on the geometry and excitation mode, these phenomena manifest as LSPR in metallic NPs or propagating SPR on planar metal films. Both effects have been extensively used in biosensing, providing real-time, label-free analysis with single-molecule sensitivity [80].

LSPR-based biosensors detect spectral shifts in NP scattering or absorption upon biomolecular binding. Their strong electromagnetic confinement enables attomolar detection of nucleic acids and proteins in complex media. Architectures such as plasmonic rulers, nanoholes, and nanopores further expand their utility, allowing precise interrogation of binding events and molecular translocation [81].

**Figure 3:**
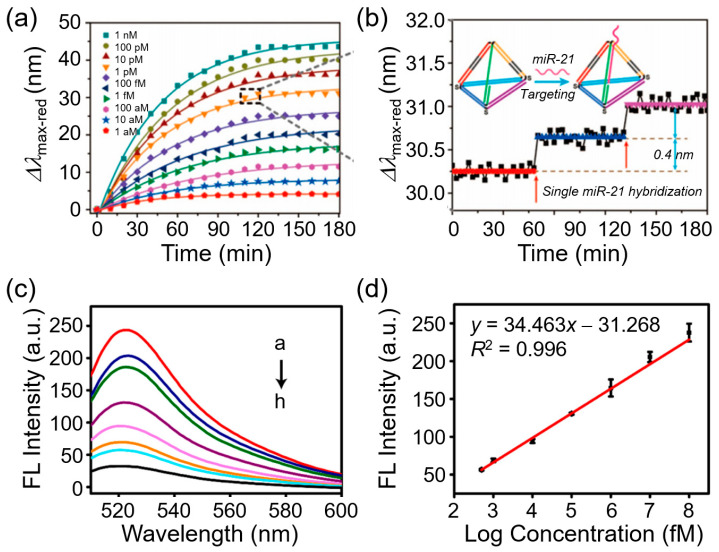
Plasmonic and CRISPR/Cas-based nanobiosensors for SMD. (**a**) Time-dependent red shift of Au@Ag nanocubes functionalized with DNA probes in response to increasing concentrations of miR-21, showing larger and faster shifts at higher concentrations. (**b**) Zoomed-in trace highlighting discrete step-like shifts from single miR-21 hybridization events, demonstrating the SMD ability of the sensor. Reprinted with permission from [82]. Copyright (2018) American Chemical Society. (**c**) Fluorescence (FL) spectra of CRISPR/Cas12a-based biosensor at various target DNA concentrations (10^8^–0 fM), showing increased emission intensity with higher DNA levels detected on Cas12a fluorescence biosensor. (**d**) Calibration curve of fluorescence intensity versus log concentration, demonstrating excellent linearity (*R*^2^ = 0.996) and reproducibility across three replicates. Reprinted with permission from [96]. Copyright (2021) Elsevier Science SA.

A key example was demonstrated by Zhang et al. by developing a smart plasmonic nanobiosensor using individual Au@Ag core–shell nanocubes (Au@Ag NCs) functionalized with tetrahedron-structured DNA for detecting microRNA-21 (miR-21) at the single-molecule level. Each hybridization event produced an average LSPR scattering spectral shift of ~0.4 nm, confirming detection at single-molecule resolution. The sensing principle was further confirmed using 3D finite-difference time-domain simulations. As shown in Figure 3a,b, this sensor exhibited time-dependent spectral shifts with increasing miR-21 concentration and stepwise spectral jumps corresponding to single hybridization events, thereby demonstrating its SMD ability. In addition, the system enabled the real-time monitoring of miR-21 with attomolar sensitivity across a broad dynamic range (1 aM–1 nM) and was further extended to perform DNA-based logic operations and biomemory assays using miR-21, KpnI, and StuI as inputs [82].

Another powerful plasmonic phenomenon is surface-enhanced Raman scattering, which relies on localized “hot spots” at nanogaps or roughened metallic surfaces to amplify Raman signals by factors up to 10^12^. This enables the acquisition of molecular vibrational fingerprints at the single-molecule level, thereby allowing the precise detection of proteins, DNA, and metabolites. In parallel, metal-enhanced fluorescence (MEF) has been employed to increase the excitation and emission rates of nearby fluorophores, thereby improving the fluorescence intensity and photostability [4,83]. Emerging approaches such as plasmonic optical tweezers and nanopore–plasmon hybrids further expand sensing capabilities by combining plasmonic near-fields and mechanical or ionic readouts for single-molecule manipulation, sequencing, and conformational analysis.

Beyond fundamental principles, several experimental studies have demonstrated the diagnostic potential of plasmonic nanobiosensors. For example, Fu et al. (2023) developed a plasmonic tweezer platform that created a dynamic silver nanoparticle (AgNP) nanocavity for single-molecule SERS, thereby enabling the real-time observation of intrinsically disordered proteins under physiological conditions [84]. Zhao et al. (2023) introduced a bowl-shaped plasmonic nanopore that concentrated near-infrared excitation into a 3 nm hotspot, allowing the Raman-based identification of the DNA strands and the discrimination of nucleotide sequences during translocation [85]. More recently, Macchia et al. (2025) demonstrated a plasmonic affinity SPR biosensor capable of detecting proteins and nucleic acids at ~10^−20^ M, equivalent to a single molecule in 0.1 mL of human serum, within 1 h [86].

Plasmonic nanobiosensors, including LSPR, SPR, SERS, MEF, and hybrid designs, provide a powerful way to probe biomolecular interactions at the single-molecule level. Their plasmon-enhanced near-fields make them well suited for coupling with SRM, where fluorescence amplification is combined with nanoscale localization. Demonstrations such as STED with gold nanorods and MINFLUX with plasmonic substrates [87,88] have primarily focused on imaging, highlighting the synergistic potential of plasmonic sensing and SRM. Exploring these approaches to nanobiosensor platforms can open new ways for ultrasensitive, spatially resolved, and dynamic SMD. However, plasmonic biosensors often exhibit batch-to-batch variability in nanostructure fabrication and relatively high costs, while inconsistent sensitivity reports highlight the need for protocol harmonization to achieve reliable clinical translation.

### 3.4. CRISPR-Based Biosensors

CRISPR/Cas systems show high potential in biosensing because of their inherent nonspecific collateral cleavage properties upon target sequence recognition [89,90,91,92]. Recently, these systems have been integrated with nanomaterials for improved performance. For example, Manganese dioxide (MnO_2_) nanoflowers were incorporated in CRISPR/Cas12a to examine intracellular monitoring via miRNA detection [93]. In another study, a colorimetric biosensor for African swine fever virus (ASFV) was developed by integrating AuNPs and magnetic beads with CRISPR/Cas12a [94]. Moreover, a manganese ion Mn^2+^-activated CRISPR/Cas12a system was advanced, where metal ions improved fluorescent signal amplification for carbaryl insecticide detection [95].

#### 3.4.1. CRISPR/Cas-Based Fluorescent Biosensors

The sensitivity of CRISPR/Cas-based nanobiosensors can be enhanced by integrating CRISPR/Cas systems with plasmonic nanomaterials, which generate fluorescent or optical signals upon biomarker recognition. In a previous study, a CRISPR/Cas12a fluorescent nanobiosensor was fabricated using the plasmonic quenching properties of graphene oxide (GO) and AuNPs [76]. 6-carboxyfluorescein (FAM)-labeled DNA probes designed as single-stranded DNA (ssDNA), double-stranded DNA (dsDNA) with single-stranded overhangs, or hairpin structures were immobilized on the nanomaterial surfaces. In the absence of the target DNA, fluorescence was quenched via FRET. Upon hybridization and target recognition, Cas12a was activated and cleaved the ssDNA segment of the probe, releasing the fluorophore and restoring fluorescence. As shown in Figure 3c,d, the biosensor achieved an impressive LOD of 134 fM for nucleic acid detection. The GO exhibited stronger quenching and higher fluorescence recovery than the AuNPs, and the staggered dsDNA probes improved the cleavage efficiency by reducing steric hindrance [96]. The fluorescence spectra demonstrated recovery upon target recognition, with the emission intensity increasing proportionally with the DNA concentration; the calibration curve exhibited excellent linearity (*R*^2^ = 0.996) and reproducibility across multiple replicates. These results highlight the strong potential of CRISPR/Cas-based fluorescent nanobiosensors for highly sensitive and quantitative nucleic acid diagnostics.

#### 3.4.2. CRISPR/Cas-Based Nonfluorescent Biosensor

Alongside fluorescent biosensor research, highly sensitive SERS-based biosensors have been developed by combining nanomaterials such as Au-coated magnetic NPs and Au core–satellite nanoclusters. In a recent study, Choi et al. developed a CRISPR/Cas12a-based SERS biosensor for viral DNA detection, eliminating the need for amplification by combining a SERS-active nanoarray with AuNPs compactly loaded with Raman probes. Specifically, a triangular Au nanoflower array with strong SERS activity was used to enhance the Raman signals many times via electromagnetic and chemical enhancement mechanisms. Raman probe-labeled AuNPs were immobilized on the nanoflower array using ssDNA linkers. Upon recognition of the target viral DNAs [hepatitis B virus (HBV) and human papillomavirus types 16 and 18 (HPV16 and HPV18)], activated CRISPR/Cas12a cleaved the ssDNA, thereby releasing the probe-modified AuNPs and considerably reducing the Raman intensity. The SERS biosensor achieved attomolar sensitivity without relying on nucleic acid amplification [97]. Despite their programmability and high sensitivity, CRISPR-based nanobiosensors still face key challenges, including off-target cleavage, occasional reliance on amplification steps, and inconsistent reproducibility across assay formats. Integration with SRM could, in principle, enable nanoscale validation of cleavage and binding interactions; however, the current complexity of assay design and the early developmental stage of these systems limit their translational potential. Achieving robust control over off-target activity and establishing truly amplification-free detection schemes will be critical for advancing CRISPR-based biosensors from exploratory proof-of-concept studies to practical diagnostic implementation.

### 3.5. Fluorescent Nanoprobe–SRM Platforms

Advances in fluorescence imaging techniques such as STED, PALM, and STORM have overcome the diffraction limit of conventional optics, enabling nanometer-scale resolution of biological nanostructures. Along with confocal and total internal reflection fluorescence (TIRF) microscopy, these techniques have become fundamental tools for single-molecule biosensing [98].

Although we briefly discuss key SRM techniques (e.g., STORM, PALM, STED, MINFLUX, and DNA-PAINT), an in-depth description of their principles is beyond the scope of this review. Comprehensive reviews are available in the literature [98,99,100,101]. Importantly, fluorescence-based assays are particularly compatible with SRM platforms because they allow direct visualization of molecular interactions with sub-diffraction accuracy, making them one of the most potent modalities for integrating nanobiosensors with advanced imaging [100,101].

QDs, which are extensively used in DNA-PAINT strategies as discussed in Section 3.2, also serve as versatile fluorescent nanoprobes for SRM-enabled immunoassays. Their high brightness, photobleaching resistance, and tunable emission spectra make them ideal for SMD, multiplexing, and long-term tracking in complex biological environments [99].

#### 3.5.1. Conventional Single-Molecule Immunoassays with SRM

Within the broad category of nanobiosensors, nanoimmunosensors form a vital subclass that exploits the specific binding between antibodies and antigens to detect biomolecules such as hormones, drugs, and proteins [102]. Immunoassays have long been used for protein quantification in clinical diagnostics and research. They have been adapted to diverse detection platforms, including optical and electrochemical systems. By incorporating nanopores or SRM, several studies have pushed LODs down to the zeptoliter (10^−21^ L) scale.

For example, Lu et al. developed an immunobiosensor to monitor anti-BSA (immunoglobulin G) IgG binding to BSA on an indium tin oxide surface. Using direct STORM (dSTORM) imaging, they visualized individual antibody–antigen binding events with superior spatial resolution and sensitivity compared with conventional microscopy [8].

#### 3.5.2. Advanced Nanoimmunosensors

A leading contributor to the integration of immune biosensors and SRM is Kang and colleagues, who have developed a series of platforms combining nanostructured chips, QD probes, and advanced optical imaging for ultrasensitive biomarker detection. Their work illustrates how coupling nanoscale architectures and SRM enables precise spatial localization, exceptional sensitivity, and high multiplexing ability.

Representative examples are as follows:

(1) Super-resolution QD-linked immunosorbent sandwich assay (srQLISA), which enabled the ultrasensitive detection of hypoxia-inducible factor 1-alpha (HIF-1*α*) with a LOD of 16 zM, approximately 10^6^-fold more sensitive than conventional ELISA [103].

(2) Super-resolution multispectral nanoimmunosensor (srMINI), which enabled the simultaneous detection of carcinoembryonic antigen (CEA), C-reactive protein (CRP), and alpha-fetoprotein (AFP) at zeptomolar concentrations, achieving over 10^8^-fold higher sensitivity than commercial ELISA kits [104].

(3) Three-dimensional TIRF-based super-localization platform, which quantified thyroid-stimulating hormone (TSH) with yoctomole sensitivity (~54 molecules); the result was validated in human serum [105].

(4) Beyond cancer biomarkers, SRM-enabled platforms have also been applied to food safety and small-molecule detection, demonstrating zepto- to yoctomolar LODs [106,107].

Fluorescence-based nanobiosensors integrated with SRM demonstrate the merging of sub-diffraction imaging and nanoscale sensing to achieve single-molecule resolution, multiplexing, and quantitative analysis. This integration not only advances our fundamental understanding of biomolecular interactions but also accelerates the development of clinically relevant diagnostic technologies. However, fluorescent nanoprobe–SRM platforms face several challenges, including photobleaching of conventional dyes, the relatively high cost of QDs and other engineered probes, and limited photostability during long-term live-cell tracking. Multiplexing capacity is further constrained by spectral overlap, and reproducibility across laboratories remains a concern. These limitations highlight the need to develop highly robust and standardized fluorescent nanoprobes before widespread clinical translation can be realized.

**Figure 4:**
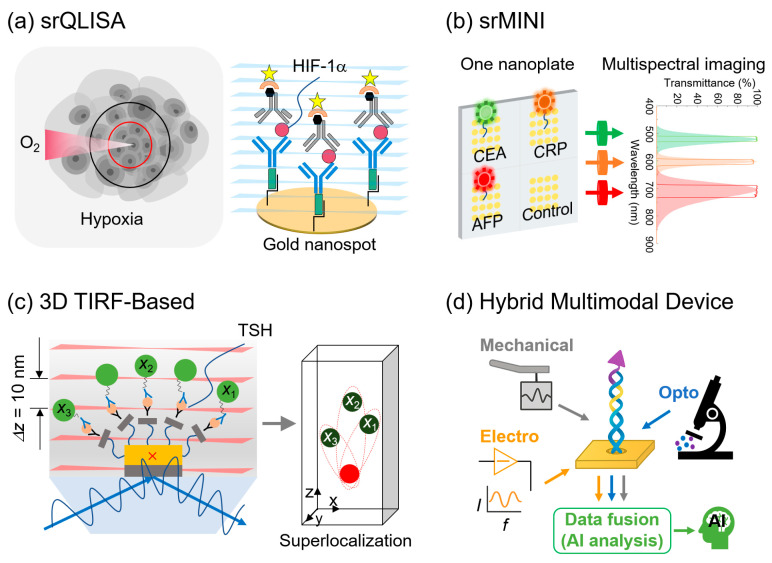
SRM-enabled nanoimmunoassays and hybrid multimodal platforms. (**a**) srQLISA for hypoxia biomarker detection (HIF-1*α*) using gold nanospot substrates. Reprinted with permission from [103]. Copyright (2022) American Chemical Society. (**b**) srMINI enabling simultaneous detection of multiple tumor markers (CEA, CRP, AFP) on a single nanoplate. Reprinted with permission from [104]. Copyright (2024) American Chemical Society. (**c**) Three-dimensional TIRF-based super-localization platform for ultrasensitive quantification of TSH with nanoscale axial resolution (Δz ≈ 10 nm). Reprinted with permission from [105]. Copyright (2023) Elsevier Science SA. (**d**) Schematic of a hybrid multimodal device integrating an optoelectromechanical (OEM) sensing platform. The device combines optical (Opto), electrical (Electro), and mechanical (Mechanical) signal transduction pathways for multidimensional single-molecule analysis. A plasmonic nanopore enables optical scattering/emission detection, electrical current modulation, and mechanical displacement sensing using cantilevers or piezoelectric MEMS elements. All signals are integrated via a unified data-fusion module employing AI analysis, achieving high-resolution and multimodal biosensing performance. Abbreviations: srQLISA, super-resolution QD-linked immunosorbent sandwich assay; srMINI, super-resolution multispectral imaging nanoimmunosensor; 3D TIRF, three-dimensional total internal reflection fluorescence; CEA, carcinoembryonic antigen; CRP, C-reactive protein; AFP, alpha-fetoprotein; HIF, hypoxia-inducible factor; TSH, thyroid-stimulating hormone; *I*, current intensity; *f*, frequency; MEMS, microelectromechanical systems.

Figure 4 summarizes representative examples of SRM-enabled nanoimmunoassays and hybrid multimodal devices. In particular, the srQLISA platform uses gold nanospot substrates for ultrasensitive detection of hypoxia biomarkers such as HIF-1*α*, achieving LODs approximately six orders of magnitude lower than those of conventional ELISA. The srMINI system further extends this ability to multiplexed profiling, enabling simultaneous quantification of tumor markers, including CEA, CRP, and AFP, on a single nanoplate. In the 3D domain, a 3D TIRF-based super-localization assay achieved the yoctomole-level quantification of TSH with an axial resolution of ~10 nm, validating its clinical utility in human serum. Beyond immunoassays, hybrid multimodal platforms that integrate plasmonic nanopores, optoelectromechanical transduction, and SRM readouts have been proposed to improve sensitivity, reduce false positives, and provide orthogonal confirmation of single-molecule events. These platforms demonstrate how coupling nanostructured immunoassays and multimodal devices with SRM can unlock unprecedented sensitivity, multiplexing, and reliability in molecular diagnostics.

### 3.6. Hybrid and Multimodal Devices

To improve sensitivity and specificity, researchers have developed hybrid nanobiosensors that integrate multiple sensing principles within a single platform. A promising direction involves merging optical and electrical modalities. For example, plasmonic nanopore sensors can concurrently monitor plasmonic resonance shifts and ionic current changes, thereby providing dual-modality confirmation of single-molecule events. This dual readout considerably improves confidence in signal assignment by reducing false positives and enabling more robust kinetic analysis [108].

As discussed in Section 3.1, mechanical motion can be linked to electronic transduction. In a recent design, DNA probes were immobilized on a GFET and driven by an alternating electric field, converting hybridization events into frequency-domain electrical signatures. This opto-electro-mechanical fusion strategy achieved sub-femtomolar sensitivity and ultra-high specificity in DNA detection, demonstrating the potential of hybrid modalities to expand beyond conventional current–voltage readouts [68]. Similarly, a one-shot dual-detection SRM imaging method was developed to monitor spatiotemporal catalytic activity variations on plasmonic gold NPs, providing real-time insights into nanoscale reaction dynamics [109].

Hybridization of distinct sensor platforms can also produce synergistic effects. For example, embedding a plasmonic LSPR chip in a photonic microcavity dramatically enhances optical signal strength for on-site pathogen and EV detection in complex biological samples [71]. Similarly, combining microfluidic confinement and plasmonic or electrochemical transducers enables real-time monitoring of rare analytes under controlled reaction conditions [110].

Looking forward, the incorporation of microfluidics is expected to play a vital role in next-generation hybrid systems, facilitating precise sample handling, digital compartmentalization, and efficient analyte trapping. These multimodal platforms are increasingly integrating AI-driven data fusion to jointly analyze optical, electrical, and mechanical signals, thereby extracting richer information from single events. Collectively, microfluidic- and AI-enhanced multimodal biosensors are advancing toward real-world POC diagnostics by combining complementary modalities for unmatched sensitivity, reproducibility, and analytical depth [106].

Importantly, hybrid biosensors also provide unique opportunities for SRM integration. For example, correlative SRM–electrochemical platforms directly align STORM-based fluorescence maps with electrochemical readouts at nanostructured electrodes. Similarly, plasmonic nanopore–SRM hybrids enable simultaneous ionic current measurements and super-resolved optical detection, providing dual confirmation of single-molecule translocation events [111,112]. These multimodal SRM-integrated systems not only reduce false positives but also improve reproducibility and provide a comprehensive view of biomolecular dynamics at the single-molecule level.

However, multimodal integration comes with certain drawbacks. The combination of optical, electrical, and mechanical modalities increases fabrication complexity and cost, often requiring custom instrumentation that complicates interlaboratory standardization. Scalability remains a significant barrier, as most multimodal architectures rely on intricate nanostructures and alignment-sensitive components. Developing simplified and standardized fabrication schemes will be essential to transition these systems from research prototypes to clinically deployable diagnostic tools.

## 4. Integration of Nanobiosensors and SRM

The convergence of nanobiosensors and SRM represents a transformative step in single-molecule diagnostics. The integration of nanobiosensors with SRM is based on pioneering microscopy innovations such as STED [19], PALM [20], and STORM [21], which first achieved sub-diffraction resolution. These breakthroughs have laid the foundation for combining biosensing sensitivity with nanoscale imaging precision. Although modality-specific examples of electrochemical, optical, plasmonic, and hybrid nanobiosensors have been introduced in Section 3, a cross-cutting synthesis is presented in this section. We detail how these platforms benefit from SRM integration and the unique opportunities this combination creates.

### 4.1. Synergistic Signal Amplification and Spatial Resolution

SRM methods such as STORM, PALM, STED, MINFLUX, and DNA-PAINT overcome the diffraction limit of conventional optics, achieving resolutions down to 10–20 nm or even below 5 nm. When coupled with the ultra-sensitivity of nanobiosensors, SRM approaches enable direct observation, precise localization, and multiplex quantification of biomolecular events that were previously undetectable.

For example, plasmonic near-fields not only improve the fluorescence intensity but also complement the ability of SRM to localize single fluorophores with nanometer precision. Similarly, electrochemical biosensors gain new dimensions of correlative analysis when paired with SRM, allowing simultaneous mapping of molecular interactions and electrical signals at the nanoscale. This dual readout reduces false positives and provides mechanistic insights that cannot be achieved using either technique alone. A representative breakthrough is the development of srQLISA, which achieved ultrasensitive detection HIF-1*α* at zeptomolar concentrations—several orders of magnitude lower than conventional ELISA. This performance was enabled by combining nanoplasmonic amplification with SRM localization precision, a capability unattainable by nanobiosensors alone [104]. In addition, plasmonic nanoprobes based on gold nanorods have been successfully combined with STED microscopy to enhance fluorophore photostability and localization precision, demonstrating synergistic benefits beyond either approach individually [82].

### 4.2. Multiplexing and Spatiotemporal Profiling

Another important benefit lies in multiplexing and spatiotemporal profiling. SRM techniques such as DNA-PAINT and multicolor STORM enable nanometer-level discrimination of multiple biomarkers, and nanobiosensors extend this ability by providing zepto- to yoctomolar LODs. This integration has already enabled ultrasensitive assays such as srQLISA and srMINI, which outperform conventional ELISA by several orders of magnitude. This approach has further been extended to a fourplex nanoimmunosensor for the simultaneous quantification of thyroid hormones at yoctomole sensitivity, highlighting the multiplexing potential of SRM-enabled nanoimmunosensors [113]. In addition, current 3D TIRF- and MINFLUX-based nanobiosensors allow real-time tracking of biomolecules in live-cell environments, bridging the gap between in vitro detection and physiologically relevant conditions. To address these limitations, a four-dimensional cuboid multiangle light-sheet SRM imaging approach has been introduced, which minimizes phototoxicity and imaging artifacts while maintaining nanoscale resolution in live-cell environments [114]. Beyond these approaches, six-dimensional tracking of anisotropic NPs in live cells has also been demonstrated using multifunctional light-sheet nanoscopy, further expanding the spatiotemporal profiling capabilities of SRM-enabled nanobiosensors [115]. Another key advance is srMINI, which can simultaneously detect multiple clinically relevant biomarkers, such as CRP, CEA, and AFP at zeptomolar concentrations. Integration with SRM overcomes spectral overlap and crosstalk limitations inherent to nanobiosensor-only platforms, enabling multiplexed detection with nanoscale spatial discrimination [104]. Similarly, MINFLUX combined with plasmonic substrates enables nanometer-to-subnanometer tracking of fluorescent molecules with minimal photon flux, demonstrating that integration achieves resolution beyond what nanobiosensors alone can provide [88].

### 4.3. Nanostructure-Assisted SRM

In addition to direct nanobiosensor–SRM hybrids, recent work on nanostructure-assisted SRM has opened a complementary route to improve imaging performance. These studies show how rational nanomaterial design can reshape local optical fields, suppress background scattering, and raise spatial resolution. They are not biosensors in the strict sense, but they help explain why materials engineering matters for reproducibility and signal stability.

Table 1 summarizes representative nanobiosensor detection methods—electrochemical, optical, plasmonic, hybrid, and immunoassay-based—that have been combined with SRM. Each one exhibits distinct advantages: electrochemical sensors enable label-free, molecular-specific detection; optical and plasmonic approaches provide high sensitivity and compatibility with fluorescence imaging; and hybrid formats combine multiple signals for multiplexed assays. However, each also has limitations: electrochemical devices suffer from electrode fouling and batch-to-batch variation, fluorescence systems face photobleaching and alignment issues, and hybrid devices encounter cost and scalability challenges. These strengths and weaknesses illustrate where SRM integration adds the most value—for example, in nanoscale mapping, multichannel detection, and orthogonal validation—and where further engineering is needed before such systems can be routinely applied as diagnostic tools. Overall, nanostructure-assisted SRM demonstrates how materials science continues to push the limits of resolution while offering design principles for reproducible and clinically deployable nanobiosensors.

Furthermore, the integration of nanobiosensors with SRM has opened new opportunities for nanoscale exosome profiling. By coupling plasmonic nanostructures with SRM imaging, researchers have achieved direct visualization and molecular classification of single exosomes, enabling the distinction between tumor-derived vesicles and normal ones [72,116,117]. Such morphological and compositional resolution is not attainable with nanobiosensors alone.

Overall, integration enables breakthroughs in sensitivity, multiplexing, and nanoscale resolution that cannot be achieved by nanobiosensors alone. The advantages and limitations of these integrated approaches are summarized in Table 2, which provides a direct comparison between nanobiosensor-only devices and nanobiosencor–SRM-integrated platforms in terms of sensitivity, LOD, spatial resolution, multiplexing capability, and clinical translational potential.

### 4.4. Technical Challenges and Opportunities in SRM Integration

Despite the obvious benefits of combining nanobiosensors and SRM, several technical challenges remain. SRM techniques require highly controlled sample preparation and precise calibration to avoid artifacts, which become even more demanding when nanostructured sensors are involved. In addition, multiplexing remains difficult. Although spatial separation strategies, such as the srMINI platform, help reduce spectral overlap, modified nanostructures can still cause crosstalk. Furthermore, prolonged SRM illumination induces photobleaching and phototoxicity, thereby limiting long-term tracking.

On the biosensor side, reproducibility is a persistent issue, because nanomaterial variability typically affects performance across experiments. However, these challenges create opportunities for innovation. Highly photostable fluorophores and hybrid plasmonic–fluor nanoprobes may reduce photobleaching, and deep learning-based SRM reconstruction can enhance resolution and suppress artifacts. In addition, integrating SRM-enabled nanobiosensors and automated microfluidics provides standardized sample handling and higher throughput, facilitating the development of additional reproducible assays. Addressing these integration-level challenges is essential for reliable SMD. Although combining nanobiosensors with SRM clearly enhances sensitivity, resolution, and multiplexing capabilities, it introduces practical limitations such as phototoxicity, photobleaching, high instrumentation costs, and limited scalability for large-scale applications. These tradeoffs indicate that, despite the notable advances in SRM-enabled nanobiosensors, clinical translation will require careful balancing of enhanced analytical performance with feasibility and accessibility. Moreover, SRM-enabled nanobiosensors have been applied to track the real-time catalytic turnover of single enzymes, revealing kinetic heterogeneity that is obscured in ensemble measurements. This level of functional insight—exemplified by a one-shot dual-detection SRM approach on plasmonic nanoparticles—demonstrated that integrating nanobiosensors with SRM enables breakthroughs beyond the capabilities of conventional biosensors [102].

### 4.5. Comparison with Previous Reviews (2018–2025)

Over the past 5–7 years, several reviews have discussed nanobiosensors or SRM; however, their focus differs substantially from this study.

2018–2020: Reviews primarily addressed plasmonic and electrochemical nanobiosensors, emphasizing sensitivity and probe design, but did not consider nanobiosensor–SRM integration [60].

2019–2021: SRM-focused reviews highlighted methodological innovations such as STORM, PALM, and MINFLUX, demonstrating improved resolution; however, their discussion of biosensing or diagnostics was limited [100,101].

2022–2023: Some reviews discussed single-molecule biosensors and optical detection, but the emphasis remained on lowering detection limits, with minimal attention to spatial mapping or SRM-enabled assays [9,11].

2024–2025: Recent reviews mentioned hybrid nanomaterials or imaging-assisted biosensors, yet these discussions were descriptive and lacked a systematic framework for integration [10,13,14].

In contrast, the present review focuses on nanobiosensor–SRM integration as the central theme. We highlight breakthroughs achievable only through this synergy, including ultrasensitive multiplexed immunoassays (srQLISA and srMINI), nanoscale exosome profiling, and correlative electrochemical–SRM readouts. In addition, we critically examine reproducibility, scalability, and clinical translational challenges, explicitly linking integration strategies to regulatory and clinical pathways. This integration-centered perspective distinguishes our work from previous reviews and provides a forward-looking roadmap for next-generation single-molecule diagnostics.

## 5. Applications in Biomolecular Diagnostics

Nanobiosensors have emerged as powerful tools for the early and precise diagnosis of various diseases, because of their exceptional sensitivity, specificity, and compatibility with POC platforms. Their applications include infectious, neurodegenerative, oncological, viral, metabolic, and renal diseases, as summarized below.

(1) Infectious diseases: Nanobiosensors enable ultrasensitive detection of pathogen-specific biomarkers for diseases such as malaria, leishmaniasis, echinococcosis, schistosomiasis, and taeniasis. Platforms incorporating advanced nanomaterials, including AuNPs, CNTs, QDs, and GO, have achieved superior sensitivity compared with ELISA and PCR while supporting multiplexed LOC systems for broad-spectrum pathogen screening and rapid POC testing [119]. In addition, plasmonic scattering-based immunosensors have demonstrated ultrasensitive pathogen detection, with recent studies reporting single-virus sensitivity across diverse infectious agents [79,117,120].

(2) Neurodegenerative diseases: For disorders such as Alzheimer’s and Parkinson’s disease, nanobiosensors provide early diagnostic ability by detecting biomarkers such as amyloid-*β* and hyperphosphorylated tau. By leveraging the unique optical, electrical, and surface properties of nanomaterials, these platforms can be functionalized with molecular beacons to enable noninvasive, rapid, and personalized diagnostic approaches, with significant potential to revolutionize disease monitoring and clinical management [121,122].

(3) Oncological applications: Nano-enhanced biosensors based on AuNPs and other nanomaterials have facilitated the detection of key cancer biomarkers, including HER2 (breast cancer), prostate-specific antigen (PSA, prostate cancer), and AFP (liver cancer). With sub-nanogram LODs, these systems enable early cancer screening, prognosis monitoring, and more effective personalized treatment planning [123,124]. In addition, plasmonic scattering-based nanoimmunosensors have been developed for ultrasensitive detection of CEA, achieving enhanced sensitivity through transmission grating-based total internal reflection scattering microscopy [125].

(4) Viral infections: QD- or CNT-based nanobiosensors have proven particularly effective for detecting viral RNA or antigens from pathogens such as human immunodeficiency virus (HIV), HBV, and SARS-CoV-2. These platforms deliver rapid results with high sensitivity and can be integrated into portable devices, making them highly suitable for on-site testing and epidemic control [126,127,128].

(5) Metabolic and renal diseases: For diabetes management, biosensors detecting HbA1c or insulin levels enable accurate assessment of glycemic control and insulin resistance. In chronic kidney disease, biosensors targeting creatinine and cystatin C provide timely diagnostic information, supporting early intervention strategies to slow disease progression [129,130]. More recently, multidimensional spatiotemporal tracking of intracellular fucoidan using plasmon-enhanced dark-field SRM imaging has been demonstrated, offering new insights into cellular metabolism and intracellular molecular interactions at the single-molecule level [131].

Although these applications demonstrate the remarkable promise of nanobiosensors and, in particular, the combination of nanoimmunosensors and SRM for single-molecule analysis, significant challenges remain before their widespread clinical translation can be realized. Overcoming issues such as reproducibility, standardization, and integration into user-friendly platforms will define the next phase of innovation in this field. Table 3 summarizes representative nanobiosensor applications in major disease areas, including the target biomarkers, nanomaterial platforms, and diagnostic advantages. These examples demonstrate the translational potential of nanobiosensors, which extend beyond proof-of-concept demonstrations toward clinically relevant diagnostics. Beyond these disease-specific applications, SRM-enabled nanobiosensors broaden the diagnostic landscape by providing unprecedented sensitivity and spatial resolution, thereby paving the way for clinical translation.

## 6. Clinical Translation Challenges and Future Directions

Translating nanobiosensor–SRM integration technologies into clinical applications remains a notable challenge. Despite impressive proof-of-concept demonstrations, few platforms have progressed beyond preclinical validation. Standardized protocols for nanomaterial synthesis, interlaboratory reproducibility, and scalable manufacturing are still largely lacking. Moreover, most published assays rely on spiked samples rather than real patient specimens, raising concerns about clinical robustness. Addressing these fundamental gaps is critical to enable effective clinical translation of nanobiosensor–SRM systems.

Beyond integration-specific issues, broader challenges must be addressed to advance nanobiosensor–SRM platforms toward clinical translation. Standardization and reproducibility across laboratories remain critical obstacles, and the high costs associated with advanced nanomaterials and SRM instrumentation hinder scalability. Bridging the gap between proof-of-concept prototypes and deployable diagnostic devices requires not only cost-effective fabrication but also rigorous validation using physiologically relevant samples and large-scale clinical trials.

Recent developments indicate that several nanobiosensor platforms are progressing toward clinical translation. For instance, plasmonic biosensors for circulating tumor DNA (ctDNA) have undergone preclinical validation, achieving detection limits of only a few copies per milliliter of plasma [132,133]. Similarly, nanoplasmonic chips for exosome profiling have been tested on clinical samples from cancer patients, demonstrating improved classification of tumor-derived versus healthy vesicles [116,134]. In addition, Food and Drug Administration (FDA)-authorized (EUA) POC molecular diagnostic systems, such as Abbott ID NOW and Cepheid GeneXpert, illustrate that nucleic acid biosensors can achieve regulatory approval and industrial adoption, although these systems do not yet incorporate SRM integration [118,135].

Complementing these case studies, clinical translation of nanobiosensor–SRM systems requires adherence to strict regulatory frameworks, such as those of the U.S. FDA, European Medicines Agency (EMA), and Korean Ministry of Food and Drug Safety (MFDS). These frameworks demand clear demonstration of analytical validity (sensitivity, specificity, and reproducibility), clinical validity, and clinical utility. At present, large-scale adoption is limited by batch-to-batch variability, fabrication scalability, cost, and usability. Addressing these intertwined technical and regulatory challenges will be essential to advance nanobiosensor–SRM systems from proof-of-concept studies to clinically approved and commercially viable diagnostic platforms [136,137].

Future directions point to several promising solutions. Advances in scalable nanomaterial synthesis and robust sensor fabrication will be crucial for enhancing reproducibility and reducing costs. Industrial–academic partnerships may accelerate technology transfer, and AI-assisted multimodal data fusion and cloud-based workflows can streamline analysis of increasingly complex datasets. Importantly, regulatory alignment and early-stage clinical evaluation are necessary to ensure safety, efficacy, and adoption in precision medicine. In parallel, deep learning has also been applied to nanobiosensor platforms themselves, as exemplified by nanozyme-based biosensors enhanced with AI algorithms to achieve improved diagnostic sensitivity and robustness [138].

These challenges and strategies are systematically summarized in Table 4. The table categorizes the barriers into technical, reproducibility, economic, regulatory, and data-related domains. It also outlines potential solutions such as standardized protocols, photostable probe design, scalable fabrication, industrial partnerships, and AI-driven data analysis. These approaches highlight a roadmap for translating nanobiosensor–SRM platforms from laboratory research into practical, clinically viable diagnostic technologies.

## 7. Conclusions

The convergence of nanoscale biosensing architectures and advanced imaging has considerably broadened the scope of molecular diagnostics, enabling the direct visualization and quantitative analysis of biomolecular events at the single-molecule level. By leveraging the structural tunability and reactivity of engineered nanomaterials, nanobiosensors achieve remarkable gains in terms of sensitivity, selectivity, and spatiotemporal resolution. When integrated with SRM, these systems overcome the diffraction barrier, thereby enabling the precise tracking of biomolecular dynamics in complex environments with zepto- to yoctomolar LODs. Such capabilities highlight their transformative potential for early disease detection, therapeutic monitoring, and precision health strategies.

Beyond sensitivity, SRM-enabled nanobiosensors provide multiplexed profiling, real-time monitoring, and 3D mapping of molecular interactions. However, challenges such as fluorophore photostability, fabrication complexity, and the gap between laboratory prototypes and clinically deployable systems remain. Addressing these challenges requires close interdisciplinary collaboration among materials scientists, optical engineers, computational modelers, and clinicians.

Looking forward, advances in next-generation QDs, hybrid plasmonic–fluorophore probes, and photostable fluorophores are expected to mitigate photobleaching and extend observation times. AI-driven image reconstruction and multimodal data fusion will improve resolution, suppress artifacts, and accelerate analysis, and integration with automated microfluidic platforms will support high-throughput, multiplexed clinical assays. Along with standardized fabrication protocols, scalable manufacturing, early regulatory engagement, and cross-laboratory benchmarking, these advances will pave the way for clinically viable diagnostic solutions.

Importantly, this review provides a perspective not fully addressed in previous studies: the systematic integration of nanobiosensors and SRM. By consolidating progress across electrochemical, optical, plasmonic, hybrid, and immunosensing modalities and by mapping their SRM-enabled applications through comparative tables, we outline a roadmap for next-generation single-molecule diagnostic platforms that are technically advanced yet clinically translatable. Successful translation will depend on urgent priorities such as experimental standardization across laboratories, interdisciplinary partnerships among material scientists, optical engineers, clinicians, and regulatory experts, as well as early alignment with regulatory frameworks (FDA, EMA, and MFDS). Addressing these priorities will be decisive for advancing from proof-of-concept studies to clinical adoption and industrial use.

In the future, the convergence of nanobiosensing and SRM technologies is expected to reshape biomedical research and diagnostics, enabling rapid single-particle infectious disease screening, nanoscale tracking of neurodegenerative processes, and real-time multiplexed monitoring of cellular signaling interactions.

## Figures and Tables

**Table 1:** Nanobiosensor modalities and their integration with SRM.

Detection Method	Principle/Advantages	Limitations	SRM Integration	Applications
Electrochemical	· Electron transfer at electrode · Miniaturization, low cost	· Electrode fouling · Limited reproducibility	Correlative STORM–electrochemical imaging	DNA hybridization, viral antigen detection
Optical	· Light–matter interactions (fluorescence, scattering, interferometry) · High sensitivity, multiplexing	· Heterogeneous signal stability · Photobleaching · Labeling required	Protein–DNA mapping (STORM/PALM), nucleic acid multiplexing (DNA-PAINT)	miRNA assays, protein–DNA interactions
Plasmonic (SPR, LSPR, SERS, MEF)	· SPR and field enhancement · Real-time, label-free, single-molecule sensitivity	· Fabrication variability · High cost	STED (improved photostability), MINFLUX (<5 nm localization)	Protein–ligand binding, biomarker detection
Hybrid/ Multimodal	· Combines optical + electrochemical or plasmonic modalities · Reduced false positives, robust sensing	· Device complexity · Limited scalability	Nanopore–SRM dual readout	Rare analyte detection, EV analysis
Fluorescent Immunoassay	· Antigen–antibody binding with nanoprobes · High specificity, multiplex detection	· Photobleaching · Probe cost · Limited photostability for long-term tracking	srQLISA (HIF-1*α*), srMINI (tumor markers)	Ultrasensitive immunodiagnostics

**Table 2:** Comparison of nanobiosensor-only devices vs. nanobiosensor–SRM-integrated approaches.

Characteristic	Nanobiosensor-Only Devices	Biosensors Integrated with SRM Techniques
Sensitivity/LOD	Femtomolar to picomolar; limited by background noise	Attomolar to zeptomolar detection; single-molecule precision [97,98]
Spatial resolution	Diffraction-limited (~200 nm); ensemble measurements	Super-resolution (20–50 nm lateral; <10 nm in advanced SRM); nanoscale localization [82,83,109]
Multiplexing capability	Limited by spectral overlap and signal crosstalk	Expanded via spatial/temporal discrimination (e.g., srMINI multiplexed assays) [98]
Information output	Bulk signal intensity; limited structural information	Quantitative, spatial mapping, and dynamic tracking of biomolecular events
Reproducibility/ Scalability	Prone to variability in nanofabrication and assay conditions	Improved validation by correlating nanosensor signals with localization accuracy; however, complexity and cost are high
Translational Potential	Early diagnostic potential, limited clinical validation	Enhanced diagnostic confidence; case studies in ctDNA, exosomes, and FDA-authorized (EUA) POC references indicate a pathway to clinical translation [118]

**Table 3:** Nanobiosensor applications in disease diagnostics.

Disease Area	Biomarkers	Sensor Type	Key Advantages
Infectious Diseases	Malaria antigens, Leishmania DNA, SARS-CoV-2 RNA	AuNPs/CNTs/QDs/GO-based LOC	Attomolar sensitivity, multiplex POC screening
Neurodegenerative	Amyloid-*β*, Tau	Molecular beacon-functionalized nanomaterial sensors	Early, noninvasive, personalized monitoring
Oncology	HER2, PSA, AFP	AuNP-based nanoimmunosensors	Sub-ng of LOD, early screening
Viral Infections	HIV RNA, HBV antigens, SARS-CoV-2	QD/CNT-based portable sensors	High-sensitivity, rapid on-site testing
Metabolic and Renal	HbA1c, Insulin, Creatinine, Cystatin C	Electrochemical and enzyme-linked nanoplatforms	Accurate glycemic/renal monitoring

**Table 4:** Current challenges and future directions for nanobiosensor–SRM platforms.

Challenges	Future Directions	Examples/Clinical Progress
Complex sample prep and calibration	· Standardized protocols · Automated calibration	POC systems validated for SARS-CoV-2 detection (Abbott ID NOW and Cepheid GeneXpert)
Multiplexing limitations	· Spatial/spectral separation · Computational unmixing	srMINI multiplexed biomarker detection in serum (proof-of-concept)
Photobleaching and phototoxicity	· Photostable QDs · Hybrid plasmonic–fluorophore probes · Low-light SRM	Validated use of long-lifetime QDs in live-cell tracking studies
Reproducibility and standardization	· Scalable · Reproducible nanomaterial synthesis · Robust sensor fabrication	Exosome nanoplasmonic chips tested with patients’ plasma (preclinical)
High cost and clinical translation gap	· Cost-effective fabrication · Industrial–academic partnerships · Integration into regulatory science frameworks	ctDNA plasmonic sensors undergoing preclinical validation
Regulatory hurdles	· Early engagement with agencies · Large-scale clinical trials	FDA emergency-use authorization for molecular diagnostics
Data complexity	· AI-driven image reconstruction · Multimodal data fusion · Cloud-based workflows and automated pipelines	Deep learning methods applied to nanozyme-based biosensors

## Data Availability

No new data were created or analyzed in this study.

## Abbreviations

The following abbreviations are used in this manuscript:

AgNP	silver nanoparticle
AFP	alpha-fetoprotein
AI	artificial intelligence
AuNP	gold nanoparticle
BAs	biogenic amines
BSA	bovine serum albumin
CNTs	carbon nanotubes
CEA	carcinoembryonic antigen
CRP	C-reactive protein
DNA-PAINT	DNA points accumulation for imaging in nanoscale topography
dSTORM	direct stochastic optical reconstruction microscopy
ELISA	enzyme-linked immunosorbent assay
EV	extracellular vesicle
FET	field-effect transistor
GFET	graphene field-effect transistor
GO	graphene oxide
HBV	hepatitis B virus
HER2	human epidermal growth factor receptor 2
HIV	human immunodeficiency virus
HPV	human papillomavirus
IgG	Immunoglobulin G
iSCAT	interferometric scattering microscopy
ITO	indium tin oxide
LOD	limit of detection
LOC	lab-on-a-chip
LSPR	localized surface plasmon resonance
MEF	metal-enhanced fluorescence
MERS	Middle East respiratory syndrome
MIPs	molecularly imprinted polymers
miRNA	microRNA
MINFLUX	minimal photon fluxes
MXenes	transition-metal carbides/nitrides (2D nanomaterials)
NPs	nanoparticles
OECTs	organic electrochemical transistors
PALM	photoactivated localization microscopy
PC	photonic crystal
PCR	polymerase chain reaction
PEG	polyethylene glycol
POC	point-of-care
QDs	quantum dots
RT-PCR	reverse transcription polymerase chain reaction
SARS-CoV-2	severe acute respiratory syndrome coronavirus 2
SERS	surface-enhanced Raman spectroscopy
SMD	single-molecule detection
smFRET	single-molecule fluorescence resonance energy transfer
SMLM	single-molecule localization microscopy
SPR	surface plasmon resonance
srQLISA	super-resolution quantum dot-linked immunosorbent assay
SRM	super-resolution microscopy
srMINI	super-resolution multispectral imaging nanoimmunosensor
STED	stimulated emission depletion microscopy
STORM	stochastic optical reconstruction microscopy
TIRF	total internal reflection fluorescence
TSH	thyroid-stimulating hormone
WGM	whispering-gallery mode
